# Long non-coding RNA HOTAIR inhibits miR-17-5p to regulate osteogenic differentiation and proliferation in non-traumatic osteonecrosis of femoral head

**DOI:** 10.1371/journal.pone.0169097

**Published:** 2017-02-16

**Authors:** Biaofang Wei, Wei Wei, Baoxiang Zhao, Xiaxia Guo, Song Liu

**Affiliations:** 1 Department of Orthopaedic, Linyi People’s Hospital, Linyi, Shandong, China; 2 Department of Orthopaedic, First School of Clinical Medicine, Guangzhou University of Chinese Medicine, Guangzhou, China; Augusta University, UNITED STATES

## Abstract

**Background and aim:**

The biological functions of non-coding RNAs (ncRNAs) have been widely identified in many human diseases. In the present study, the relationship between long non-coding RNA HOTAIR and microRNA-17-5p (miR-17-5p) and their roles in osteogenic differentiation and proliferation in non-traumatic osteonecrosis of femoral head (ONFH) were investigated.

**Methods:**

The expression levels of HOTAIR and miR-17-5p in the mesenchymal stem cells (MSCs) derived from patients with non-traumatic ONFH and osteoarthritis (OA) were examined by real-time PCR. BMP-2 induced human MSCs from bone marrow (hMSC-BM) were used for osteogenic differentiation.

**Results:**

It was observed that the expression level of miR-17-5p was lower and the level of HOTAIR was higher in samples of non-traumatic ONFH compared with OA. HOTAIR downregulation induced by si-HOTAIR led to the increase of miR-17-5p expression and the decrease of miR-17-5p target gene SMAD7 expression. The values of osteogenic differentiation markers, including RUNX2 and COL1A1 mRNA expression and ALP activity, were also elevated by si-HOTAIR. However, the increase of these values was canceled by miR-17-5p inhibitor or SMAD7 upregulation.

**Conclusion:**

HOTAIR played a role in regulating osteogenic differentiation and proliferation through modulating miR-17-5p and its target gene SMAD7 in non-traumatic ONFH.

## Introduction

Osteonecrosis of the femoral head (ONFH) is a bone-destructive disease that mainly induced by the destruction of the blood supply and the disorder of coagulation and fibrinolysis system, ultimately leading to the collapse of femoral head. This disease commonly affects patients aged 30 to 50 years and makes a tremendous difference in the quality of life. Hormone and alcohol are the commonest causes of non-traumatic ONFH. It has been proposed that the altered osteogenic differentiation capability of mesenchymal stem cells (MSCs) contributes to the imbalance of osteonecrosis and bone regeneration, which is a critical factor in the pathogenesis of non-traumatic ONFH [1]. However, the specific molecular mechanisms of aberrant osteogenic differentiation of MSCs should be largely studied.

In recent years, researchers have focused on the biological function of the non-coding RNA (ncRNA), a RNA not translated into a protein, in various human diseases [2]. MicroRNA (miRNA) is one type of ncRNAs that is the most studied [3]. It is known that miRNAs participate in regulating gene expression to influence diverse cellular processes. Emerging evidence has showed that lots of miRNAs have been identified to participate in the process of osteogenesis, including osteoclast formation, differentiation, apoptosis, and resorption [4, 5]. The new published paper confirmed 39 differential miRNAs between non-traumatic ONFH samples and femoral neck fracture samples using miRNA microarray chip analysis [6], indicating miRNAs might be important in the pathogenesis of non-traumatic ONFH. Previous studies have showed that miR-17-5p play a regulator role in various cell proliferation and differentiation [7]. A study revealed that miR-17-5p expression was significantly lower in non-traumatic ONFH than that in osteoarthritis (OA) samples, and functioned to facilitate the proliferation and differentiation of MSCs [8]. However, how miR-17-5p is regulated in non-traumatic ONFH remains unclear.

The long non-coding RNA (lncRNA) is another very important type of ncRNAs that also behaves as a regulator in various human diseases. Recent studies have proposed that lncRNAs can target miRNA to regulate gene expression and take part in the cellular processes [9]. Homeobox transcript antisense RNA (HOTAIR) has been widely validated having an unignorable role in oncogenic progression [10]. It is identified that HOTAIR is expressed in human cartilage samples [11]. We found that HOTAIR had the complementary sequences of miR-17-5p using bioinformatics software. However, the relationship between them is not studied. In the present study, whether HOTAIR was involved in the regulation of miR-17-5p expression to exhibit a role in osteogenic differentiation and proliferation in non-traumatic ONFH was investigated.

## Materials and methods

### Subjects and specimen collection

The patients with non-traumatic ONFH (n = 15) or OA (n = 13) were recruited from the Linyi People’s Hospital. The human related study was approved by the ethics committee of Linyi People’s Hospital and written informed consent was obtained from all patients. All patients underwent surgery and a total of 5 ml bone marrow specimens from the proximal end of the femurs in each subject were collected.

### MSCs isolation

Bone marrow aspirates were diluted with 2 mM EDTA-PBS, and mononuclear cells were isolated by a Ficoll-Hypaque density gradient centrifugation. Then the cells were maintained in low-glucose Dulbecco’s modified Eagle’s medium (DMEM-LG, Invitrogen, USA) supplemented with 10% fetal bovine serum (FBS; Gibco, USA) and 1% antibiotic-antimycotic solution (Invitrogen, USA) at 37°C in an atmosphere containing 5% CO_2_. When MSCs grew to 80%–90% confluence, the cells were harvested and incubated with 0.25% trypsin for passage. Osteogenic differentiation ability of MSCs was estimated by measuring the mean percentage of the area stained with alizarin red S, and the results showed that the mean percentage of the areas in OA and non-traumatic ON were 78±9% and 54±5%, respectively. Adipogenic differentiation ability of MSCs was estimated by measuring the optical density of the Oil red O staining, and the results showed that the optical densities were 0.62±0.1 and 0.58±0.09 in OA and non-traumatic ON, respectively.

### Cell line and cell culture

Human MSCs from bone marrow (hMSC-BM) was purchased from ScienCell Research Laboratories (USA). The cells were maintained in high-glucose DMEM supplemented with 10% FBS and 1% antibiotic-antimycotic solution (Invitrogen, USA) at 37°C in an atmosphere containing 5% CO_2_. When MSCs grew to 80%–90% confluence, the cells were harvested and incubated with 0.25% trypsin for passage. For osteoblastic differentiation, the medium was replaced for fresh medium containing 10% FBS and 100 ng/ml recombinant human bone morphogenetic protein-2 (BMP-2, R&D Systems, USA) for 6 days.

### Cell transfection

In this study, several specific sequences with or without vector were used for overexpressing or inhibiting gene expression. The interference sequences for HOTAIR and SMAD7 were named as siRNA-HOTAIR (si-HOTAIR) and si-SMAD7, respectively, with si-control acting as their negative control. The sequences of the siRNA for HOTAIR were 5’-GGAGAACACUUAAAUAAGUTT-3’ (sense) and 5’-ACUUAUUUAAGUGUUCUCCTA-3’ (antisense). The sequences of the siRNA for SMAD7 were 5'-AAGATAATTCGTTCCCCCTGTCCTGTCTC-3' (sense) and 5'-AAACAGGGGGAACGAATTATCCCTGTCTC-3' (antisense). The overexpressing sequences for HOTAIR and SMAD7 constructed into adenoviral vector and were named as Ad-HOTAIR and Ad-SMAD7, respectively, with Ad-GFP acting as their negative control. Pre-negative control (NC) and NC were used as the controls of miR-17-5p mimic and miR-17-5p inhibitor, respectively. All these sequences or vectors were synthesized by Shanghai GenePharma Co., Ltd (shanghai, China) and transfected into hMSC-BM cells using the Lipofectamine 2000 reagent (Invitrogen, USA) following the manufacturer’s instructions. In brief, cells were plated in 24-well plates one day before the transfection, and cultured in medium without serum. When the cells reached 70%-90% confluence, the cells were transfected with interference sequences or adenoviral vector. At 6h after transfection, the medium was replaced by medium with serum. For some experiments, at 48h after transfection, BMP-2 was used for inducing osteoblastic differentiation.

### Real-time PCR

Total RNA from MSCs derived from patients and cultured hMSC-BM were isolated using TRIzol reagent (Invitrogen, USA) according to the manufacturer’s instructions. The complementary DNA (cDNA) was produced using M-MLV Reverse transcriptase (Invitrogen, USA) with RNA acting as a template. The quantitative analysis of miR-17-5p, HOTAIR, SMAD7, RUNX2, and COL1A1 expression was performed with QuantiTect SYBR Green RT-PCR kit (QIAGEN, USA) in triplicates by reacting with specific primers. The relative expression level of target genes was expressed as fold changes through normalization against reference gene expression using the 2^-ΔΔCt^ method. In this study, U6 and GAPDH were acted as the reference genes for miR-17-5p and HOTAIR, respectively. β-Actin was used as the reference gene for SMAD7, RUNX2, and COL1A1. The primer sequences for miR-17-5p, HOTAIR, SMAD7, RUNX2, COL1A1, U6, GAPDH and β-Actin were as follows: miR-17-5p, forward, 5'-TGCGC CAAAG TGCTT ACAGT GCA -3', reverse, 5'-CCAGT GCAGG GTCCG AGGTA TT-3; HOTAIR, forward, 5'-CATGG ATCCA CATTC TGCCC TGATT TCCGG AACC-3', reverse, 5'-ACTCT CGAGC CACAC ACACA CACAC CTACAC-3'; SMAD7, forward, 5'-AGCAG GCCAC ACACT TCAAA CT-3', reverse, 5'-CACGT TGTCT CCCCA TCTG-3'; RUNX2, forward, 5'- GGCAG GCACA GTCTT CCC-3', reverse 5'-GGCCC AGTTC TGAAG CACC-3'; COL1A1, forward, 5'-CGATG GATTC CAGTT CGAGT-3', reverse, 5'-TTTTG AGGGG GTTCA GTTTG-3'; U6, forward, 5'-CGCTT CGGCA GCACA TATAC-3', reverse, 5'-AATAT GGAAC GCTTC ACGA-3'; GAPDH, forward, 5'-GGGAG CCAAA AGGGT CAT-3', reverse, 5'-GAGTC CTTCC ACGAT ACCAA-3'; β-Actin, forward, 5'-TTGTT ACAGG AAGTC CCTTG CC-3', reverse, 5'-ATGCT ATCAC CTCCC CTGTG TG-3'.

### DNA methylation measurements

For analysis of DNA methylation levels for miR-17-5p promotor, genomic DNA from MSCs derived from patients and hMSC-BM after the transfection of si-HOTAIR were isolated using a Genomic DNA isolation kit (BioVision, USA). Then, the collected DNA was subjected to bisulfite conversion and purification using the EZ DNA Methylation-GoldTM Kit (Zymo Research Co., USA) following the manufacturer’s protocol. PCR were performed and PCR products were analyzed by 2% agarose gel electrophoresis. For bisulphite sequencing, PCR products were cloned into TOPO TA vector and the positive clones were sequenced (Sangon Biotech Co., Shanghai, China). The percentage of methylation level of miR-17-5p promotor was calculated.

### Western blot

Total protein from hMSC-BM cells after the transfection of si-HOTAIR was isolated and quantified using RIPA Lysis Buffer and BCA Protein Assay Kit (Beyotime, China), respectively. Each equal amount of protein was run on 10% sodium dodecyl sulfate-polyacrylamide gel electrophoresis (SDS-PAGE), and then transferred to polyvinylidene fluoride (PVDF)-membranes. The membranes were blocked with 5% BSA for 2 h, and the blots were incubated with primary antibody against SMAD7 (1: 1000, Abcam, UK) overnight at 4°C, with β-Actin acting as control, then incubated with goat anti-rabbit HRP-IgG (1: 1000, Abcam, UK) for 2 h at room temperature. The bands were visualized using BeyoECL Plus ECL Kit (Beyotime, China) and images by gel image analysis system.

### ALP activity

Alkaline phosphatase (ALP) activity was used to evaluate the osteogenic differentiation capability of hMSC-BM after the different administrations. In brief, the cells were lysed with a lysis buffer containing 0.1% Triton X-100. The supernatant of lysate was used to determine the ALP activity by incubating with p-nitrophenyl phosphate (pNPP) at 37°C for 15min. Then, the absorbance was measured at a wavelength of 405 nm using a microplate reader. Total DNA concentration was determined by CyQUANT^®^ Cell Proliferation Assays (Thermo Fisher Scientific, USA). The ALP activity was normalized to total DNA concentration.

### Statistical analysis

All data were expressed as mean ± standard error (SE). The differences among three or more groups were analyzed with one-way ANOVA, and the difference between two groups was compared with independent-samples T test. *P* < 0.05 was considered as statistically significant.

## Results

### The expression levels of miR-17-5p and HOTAIR in MSCs of patients with non-traumatic ONFH or OA

**Fig 1:**
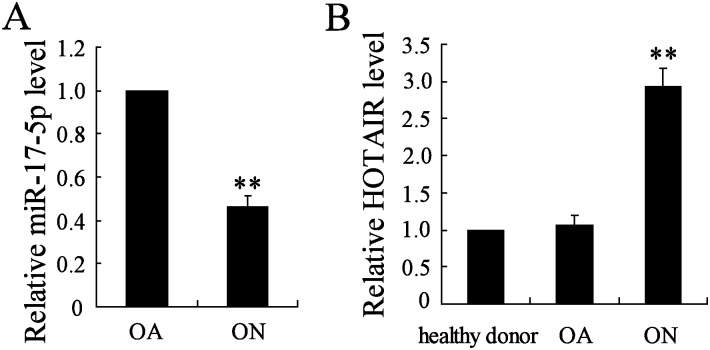
The expression levels of miR-17-5p and HOTAIR in MSCs of patients with non-traumatic necrosis of femoral head and osteoarthritis. The MSCs were isolated from patients with non-traumatic necrosis of femoral head (ONFH, n = 15), osteoarthritis (OA, n = 13) and healthy donor (n = 10), the expression levels of miR-17-5p (A) and HOTAIR (B) were detected by real-time PCR. Each sample was repeated three times. ** *P*<0.01.

We first investigated the differential expression levels of miR-17-5p and HOTAIR in MSCs derived from patients with non-traumatic ONFH, OA and healthy donor. The results showed that miR-17-5p expression was significantly lower (*P*<0.01, Fig 1A) in non-traumatic ONFH group than that in OA group, and HOTAIR expression was significantly higher (*P*<0.01, Fig 1B) in non-traumatic ONFH group than that in both OA group and healthy donor. It also observed that HOTAIR expression had no statistical difference between healthy donor and OA groups (*P*>0.01, Fig 1B).

### Epigenetic regulation influences the expression of miR-17-5p

**Fig 2:**
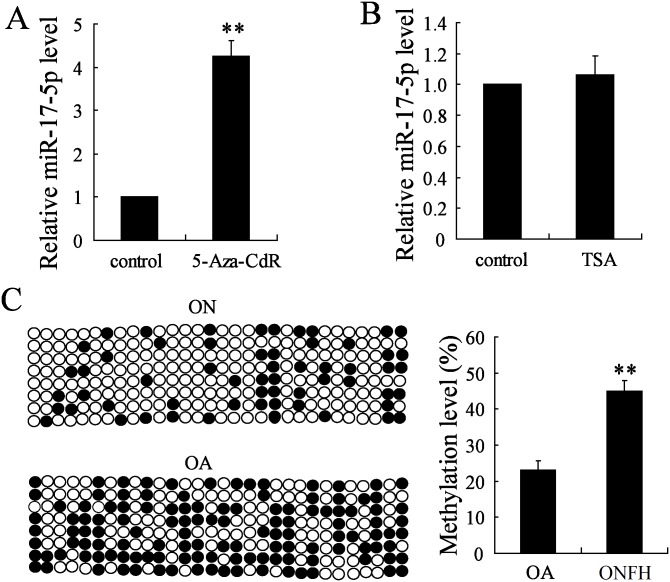
The effects of methylation and histone acetylation alteration on miR-17-5p expression regulation. The MSCs were isolated from patients with non-traumatic necrosis of femoral head (ONFH, n = 15) and osteoarthritis (OA, n = 13). The expression levels of miR-17-5p in MSCs derived from patients with non-traumatic ONFH was detected by real-time PCR after the treatment of 5 μmol/l 5-Aza-2-deoxycytidine (5-Aza-CdR, A) or 100 nmol/l Trichostatin A (TSA, B) for 48h. The DNA methylation level of miR-17-5p promotor in MSCs from two groups were determined by bisulphite sequencing (C). Each sample was repeated three times. ** P<0.01.

To explore the potential mechanism that contributes to regulate miR-17-5p expression, the epigenetic regulation of miR-17-5p expression was then determined. The MSCs derived from non-traumatic ONFH patients were treated with 5-Aza-CdR, an epigenetic modifier results in DNA demethylation, or TSA, a histone deacetylase inhibitor, and it was observed that miR-17-5p expression was markedly increased by 5-Aza-CdR (*P*<0.01) but not influenced by TSA (*P*>0.05) (Fig 2A and 2B), indicating that the aberrant methylation might be a contributor for miR-17-5p expression regulation. We then analyzed the differential methylation levels of miR-17-5p promotor in MSCs between non-traumatic ONFH and OA groups. The results showed that DNA methylation level of miR-17-5p promotor in non-traumatic ONFH group was significantly higher compared with the OA group (*P*<0.01) (Fig 2C).

### HOTAIR downregulation increased miR-17-5p expression

**Fig 3:**
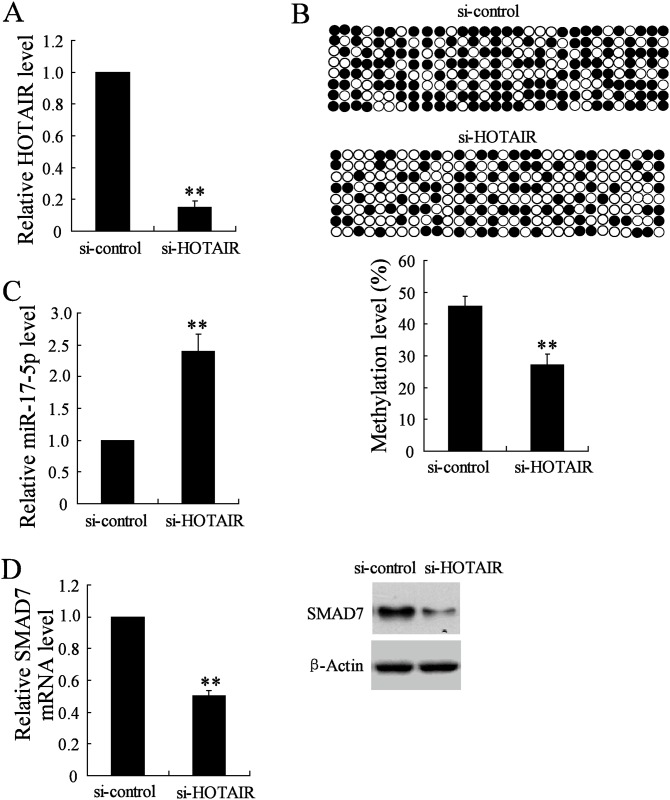
The effects of HOTAIR downregulation on the expression levels of miR-17-5p and its target gene. The MSCs were isolated from patients with non-traumatic necrosis of femoral head (ONFH), and transfected with siRNA-HOTAIR (si-HOTAIR) or siRNA-control (si-control). The HOTAIR expression level was measured by real-time PCR (A). The DNA methylation level of miR-17-5p promoter was determined by bisulphite sequencing assay (B). The miR-17-5p expression level was measured by real-time PCR (C). The SMAD7 expression in mRNA and protein levels was measured by real-time PCR and western blot (D). n = 3, each sample was repeated three times. ** *P*<0.01.

The role of HOTAIR in the regulation of miR-17-5p expression was then investigated. Real-time PCR first confirmed that si-HOTAIR transfection induced the significant decrease of HOTAIR expression (Fig 3A). The following experiments showed that downregulation of HOTAIR contributed to the reduced DNA methylation level of miR-17-5p promotor (Fig 3B) and the elevated miR-17-5p expression (Fig 3C). In addition, the mRNA and protein expression levels of SMAD7, a target gene of miR-17-5p, were also decreased by the treatment of si-HOTAIR (Fig 3D).

### HOTAIR expression was decreased during the process of osteogenic differentiation

**Fig 4:**
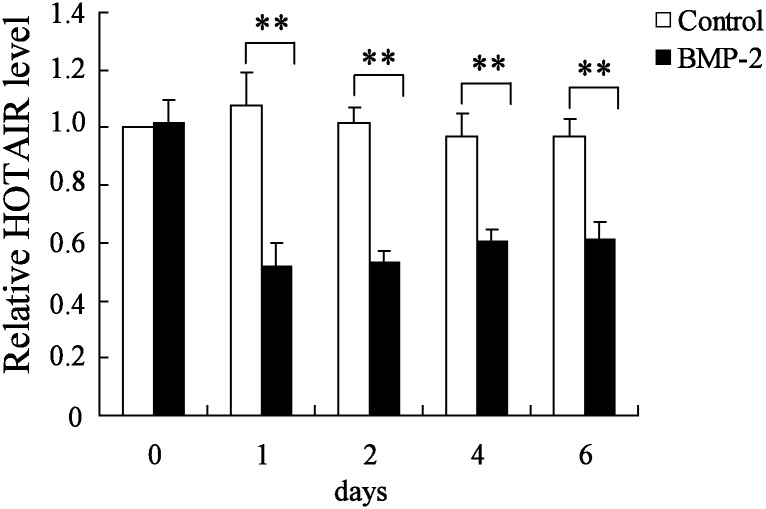
HOTAIR expression in BMP-2-induced osteoblastic differentiation. HOTAIR expression level was detected by real-time PCR in human mesenchymal stem cells from bone marrow (hMSC-BM) cell line after the treatment of osteoblastic-inductive BMP-2 for six days. n = 3, each sample was repeated three times. ** *P*<0.01.

To determine the potential role of HOTAIR during the process of osteogenic differentiation, the HOTAIR expression in BMP-2 induced osteogenic differentiation was measured. The results showed that HOTAIR expression was significantly decreased in stromal cell line HMSC-bm after the treatment of BMP-2, a well-known inducer of osteoblast differentiation (Fig 4). These data suggested that HOTAIR was probably an important factor during the process of MSC differentiation.

### HOTAIR regulates osteogenic differentiation markers

**Fig 5:**
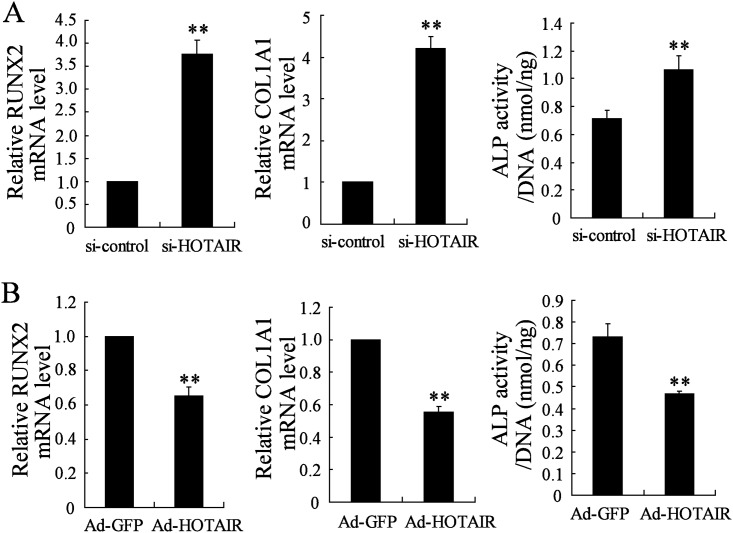
The effects of HOTAIR on osteogenic differentiation markers. Human mesenchymal stem cells from bone marrow (hMSC-BM) cell line was transfected with siRNA-HOTAIR (si-HOTAIR, with si-control acting as control, A) or Adenovirus-HOTAIR (Ad-HOTAIR, with Ad-GFP acting as control, B) for 48h, then incubated with osteoblastic-inductive BMP-2 for six days. The mRNA levels of RUNX2 and COL1A1 were detected by real-time PCR and ALP activity was measured by Kits. n = 3, each sample was repeated three times. ** *P*<0.01.

To further investigate the effects of HOTAIR on the process of osteogenesis differentiation, osteogenic differentiation markers including RUNX2, COL1A1 and ALP were evaluated in hMSC-BM cells after the transfection of si-HOTAIR or Ad-HOTAIR for inhibiting or up-regulating HOTAIR expression. As shown in Fig 5, down-regulation of HOTAIR induced the significant increase in mRNA expression levels of RUNX2 and COL1A1 and in ALP activity (Fig 5A). While HOTAIR upregulation led to the opposite results (Fig 5B).

### HOTAIR regulates osteogenic differentiation markers through mediating miR-17-5p expression

**Fig 6:**
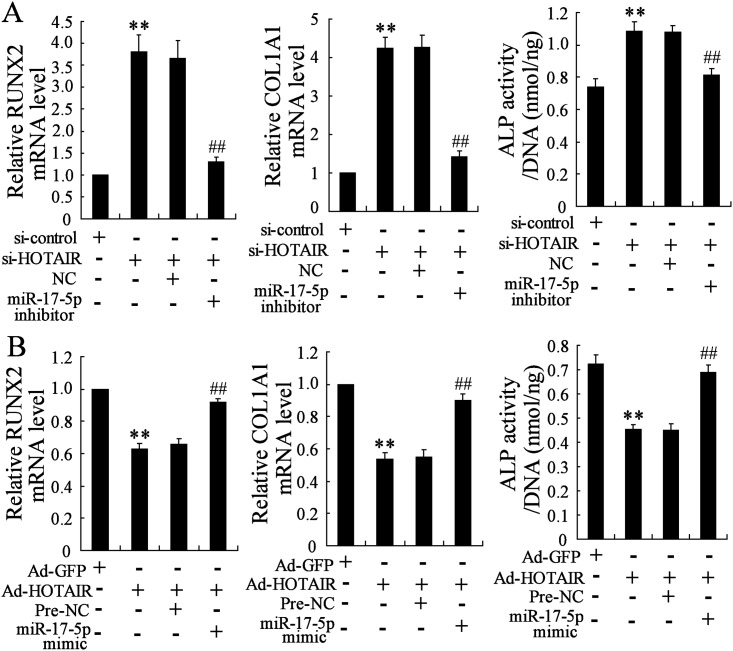
HOTAIR regulates osteogenic differentiation markers through mediating miR-17-5p expression. Human mesenchymal stem cells from bone marrow (hMSC-BM) cell line was treated with co-transfection of siRNA-HOTAIR (si-HOTAIR) and miR-17-5p inhibitor, with si-control and NC acting as controls, respectively (A) or co-transfection of Adenovirus-HOTAIR (Ad-HOTAIR) and miR-17-5p mimic, with Ad-GFP and Pre-NC acting as controls, respectively (B) for 48h, then incubated with osteoblastic-inductive BMP-2 for six days. The mRNA levels of RUNX2 and COL1A1 were detected by real-time PCR and ALP activity was measured. n = 3, each sample was repeated three times. ** *P*<0.01.

Whether HOTAIR participates in the regulation of osteogenic differentiation that mediates miR-17-5p expression remains unclear. In this experiment, we observed that miR-17-5p inhibitor canceled the increase in mRNA expression levels of RUNX2 and COL1A1 and in ALP activity which were induced by si-HOTAIR (Fig 6A). On the other hand, the decrease in RUNX2 and COL1A1 mRNA expression levels and ALP activity induced by Ad-HOTAIR was also reversed by miR-17-5p mimic (Fig 6B). These data indicated that HOTAIR regulated osteogenic differentiation through mediating miR-17-5p expression.

### HOTAIR regulates osteogenic differentiation markers through mediating SMAD7 expression

**Fig 7:**
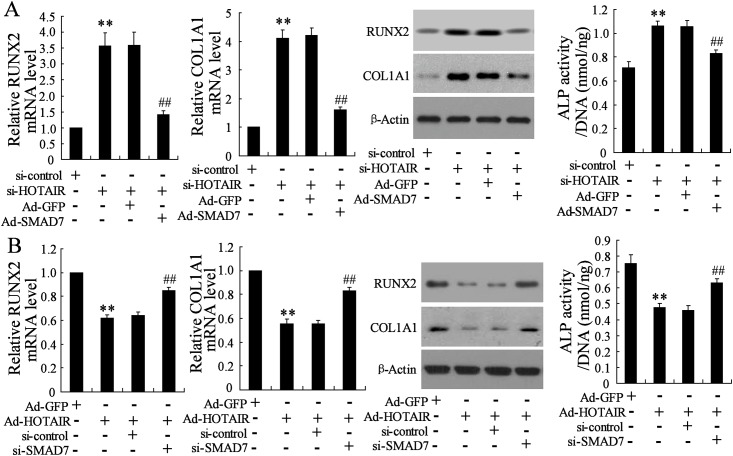
HOTAIR regulates osteogenic differentiation markers through mediating SMAD7 expression. Human mesenchymal stem cells from bone marrow (hMSC-BM) cell line was treated with co-transfection of siRNA-HOTAIR (si-HOTAIR) and Adenovirus-SMAD7 (Ad-SMAD7), with si-control and Ad-GFP acting as controls, respectively (A) or co-transfection of Ad-HOTAIR and si-SMAD7, with Ad-GFP and si-control acting as control, respectively (B) for 48h, then incubated with osteoblastic-inductive BMP-2 for six days. The mRNA and protein levels of RUNX2 and COL1A1 were detected by real-time PCR and western blot, and ALP activity was measured. n = 3, each sample was repeated three times. ** *P*<0.01.

It has been reported that SMAD7 is a target gene of miR-17-5p that modulates osteoblastic differentiation. The results mentioned above confirmed that HOTAIR regulated osteogenic differentiation markers through mediating miR-17-5p expression. We then evaluated whether SMAD7 was involved in this process. As shown in Fig 7A, Ad-SMAD7 canceled the increase in mRNA as well as protein expression levels of RUNX2 and COL1A1 and in ALP activity which were induced by si-HOTAIR (Fig 7A). On the other hand, the decrease in RUNX2 and COL1A1 expression levels and ALP activity induced by Ad-HOTAIR was also reversed by si-SMAD7 (Fig 7B). These findings suggested that SMAD7 was mediated by HOTAIR and involved in the regulation of osteogenic differentiation.

## Discussion

Previous study confirmed that miR-17-5p modulated osteoblastic differentiation and cell proliferation in non-traumatic ONFH [8]. We also observed that miR-17-5p expression was decreased in non-traumatic ONFH samples, and HOTAIR exhibited an upstream regulator for miR-17-5p to modulate the osteoblastic differentiation of MSC.

HOTAIR, the first discovered trans-acting lncRNA, has been found to be aberrantly overexpressed in many kinds of tumor tissues and cell lines such as gastric cancer, breast cancer, hepatic carcinoma, ovarian cancer and acute myeloid leukemia [12]. The functional roles of HOTAIR in tumor biology have been largely studied. In recent years, its biological functions in many other human diseases and physiological processes are also partly revealed. For example, HOTAIR is associated with the development or progression of spermiogenesis [13], aortic valve calcification [14], neurodegeneration diseases [15] and hepatitis B virus-induced liver carcinogenesis [16]. Zhou et al found that the genetic variants of HOTAIR lead to the risk of osteosarcoma [17]. Microarray analysis employed by Xing et al identified that HOTAIR was expressed in OA cartilage and its level was higher than that in normal samples [11]. However, the biological functions of HOTAIR in non- traumatic ONFH and osteogenic differentiation remains unknown. In the present study, we first reported that HOTAIR expression was significantly higher in MSCs derived from patients with non-traumatic ONFH compared with OA group, which indicated a potential role in the pathogenesis of non-traumatic ONFH. We also detected the HOTAIR expression in MSCs sample derived from patients with alcohol-induced ON (n = 10), steroid-induced ON (n = 10) and idiopathic ON (n = 10), the results showed that HOTAIR expression was significantly elevated in these three groups than that in patients with OA. In addition, there were no statistical differences of HOTAIR expression among these three ON groups. Therefore, it was proposed that HOTAIR participates in regulating osteogenic differentiation and proliferation in ONFH regardless of the etiology classification. *In vitro* experiments, during the process of osteogenic differentiation of hMSC-BM induced by BMP-2, the HOTAIR expression was observed to be decreased, further suggesting a potential function in osteogenic differentiation. The functions of HOTAIR in osteogenic differentiation markers, including RUNX2, COL1A1 and ALP, were also determined. It observed that HOTAIR down-regulation contributed to the increase of mRNA expression of RUNX2 and COL1A1 and the elevation of ALP activity in BMP-2 induced hMSC-BM. These data confirmed that HOTAIR was a regulator for osteogenic differentiation.

MiR-17-5p is located in the miR-17/92 cluster. Many studies have proved that miR-17-5p plays a key role in tumor biology, including cancer proliferation, cell migration, cell cycle progression, cell apoptosis and tumor growth [18–20]. In osteosarcoma, miR-17-5p up-regulation is more frequently occurred in specimens with advanced clinical stage, positive distant metastasis and poor response. MiR-17-5p promotes the cell proliferation of osteosarcoma in a BRCC2-dependent mechanism [21]. Li et al first reported that the expression levels of miRNA-17 family, including miR-17-5p and miR-106a were down-regulated in response to BMP2 in C2C12 samples that underwent osteoblast differentiation [22]. The decreased expression levels of miR-17-5p and miR-106a in human adipose-derived MSCs (hADSCs) underwent differentiation toward osteogenic lineages and increased levels during adipocyte differentiation were also confirmed by Li et al [23]. They also confirmed that miR-17-5p regulated osteogenic and adipogenic lineage commitment of hADSCs by directly targeting BMP2. In recent years, it demonstrated that miR-17-5p modulates osteoblastic differentiation and cell proliferation by targeting SMAD7 in non-traumatic ONFH [8], which is consistent with our findings. Fang et al found that miR-17-5p suppresses osteogenic differentiation and bone formation by targeting SMAD5 [24].

Bioinformatics software reports that HOTAIR shares the complementary sequences with miR-17-5p, indicating HOTAIR may be a regulator for the expression of miR-17-5p. The epigenetic alterations of miRNAs are important for the regulation of their expression, the study of which is a novel area in various diseases [25]. We observed that the change of miR-17-5p expression could be induced by the DNA methylation inhibitor (5-Aza-CdR) but not histone deacetylase inhibitor (TSA). Therefore, the methylation level of miR-17-5p was detected in non-traumatic ONFH and the results showed that DNA methylation level of miR-17-5p promotor was significantly higher compared with the OA group. In addition, HOTAIR downregulation contributed to the reduced DNA methylation level of miR-17-5p promotor and increased miR-17-5p expression, which proved that HOTAIR modulated the expression level of miR-17-5p. Our further experiments demonstrated the decrease of RUNX2 and COL1A1 mRNA expression levels and ALP activity induced by HOTAIR upregulation was reversed by miR-17-5p mimic, while the increased values induced by HOTAIR downregulation was also canceled by miR-17-5p inhibitor. In addition, the effects of HOTAIR upregulation/downregulation on the alterations of osteogenic differentiation related parameters were also reversed by the downregulation/ upregulation of miR-17-5p target gene SMAD7. These data indicated that HOTAIR was involved in the regulation of osteogenic differentiation through modulating the expression of miR-17-5p and its target gene SMAD7.

In conclusion, our findings demonstrated that HOTAIR was significantly higher and miR-17-5p expression was significantly lower in non-traumatic ONFH group than that in OA group. HOTAIR played a role in regulating osteogenic differentiation and proliferation through modulating miR-17-5p and its target gene SMAD7. This study proposed a new biomarker for indicating non-traumatic ONFH and providing a novel insight for understanding the potential mechanism of osteogenic differentiation in non-traumatic ONFH.
